# The Complex Interactions Between Flowering Behavior and Fiber Quality in Hemp

**DOI:** 10.3389/fpls.2019.00614

**Published:** 2019-05-16

**Authors:** Elma M. J. Salentijn, Jordi Petit, Luisa M. Trindade

**Affiliations:** Plant Breeding, Wageningen University and Research, Wageningen, Netherlands

**Keywords:** hemp, *Cannabis sativa*, short-day plant, flowering-time, phenology, sex determination, fiber development

## Abstract

Hemp, *Cannabis sativa* L., is a sustainable multipurpose fiber crop with high nutrient and water use efficiency and with biomass of excellent quality for textile fibers and construction materials. The yield and quality of hemp biomass are largely determined by the genetic background of the hemp cultivar but are also strongly affected by environmental factors, such as temperature and photoperiod. Hemp is a facultative short-day plant, characterized by a strong adaptation to photoperiod and a great influence of environmental factors on important agronomic traits such as “flowering-time” and “sex determination.” This sensitivity of hemp can cause a considerable degree of heterogeneity, leading to unforeseen yield reductions. Fiber quality for instance is influenced by the developmental stage of hemp at harvest. Also, male and female plants differ in stature and produce fibers with different properties and quality. Next to these causes, there is evidence for specific genotypic variation in fiber quality among hemp accessions. Before improved hemp cultivars can be developed, with specific flowering-times and fiber qualities, and adapted to different geographical regions, a better understanding of the molecular mechanisms controlling important phenological traits such as “flowering-time” and “sex determination” in relation to fiber quality in hemp is required. It is well known that genetic factors play a major role in the outcome of both phenological traits, but the major molecular factors involved in this mechanism are not characterized in hemp. Genome sequences and transcriptome data are available but their analysis mainly focused on the cannabinoid pathway for medical purposes. Herein, we review the current knowledge of phenotypic and genetic data available for “flowering-time,” “sex determination,” and “fiber quality” in short-day and dioecious crops, respectively, and compare them with the situation in hemp. A picture emerges for several controlling key genes, for which natural genetic variation may lead to desired flowering behavior, including examples of pleiotropic effects on yield quality and on carbon partitioning. Finally, we discuss the prospects for using this knowledge for the molecular breeding of this sustainable crop *via* a candidate gene approach.

## Introduction

Hemp (*Cannabis sativa* L.) is increasingly attractive as multipurpose crop for the sustainable production of fibers, oils, and cannabinoids (Van der Werf et al., 1996; Struik et al., 2000; Callaway, 2004; Karus and Vogt, 2004; Van der Werf, 2004; Barth and Carus, 2015; Andre et al., 2016, and references therein). As a quantitative short-day plant, photoperiod and temperature input are the key factors that determine the timing of flowering. Adaptation to the latitude of growth, characterized by a specific photoperiod and temperature regime, is very important for hemp production. Hemp flowering is inhibited in a regime of long-day photoperiod (LD) and is induced when a number of short-day photoperiods (SD) have passed (threshold ~10–12 h of uninterrupted darkness; critical photoperiod ~14–12 h of daylight). If the critical short-day is not reached within the growing season or if cultivars are very late flowering, the plants remain vegetative. Another important aspect of hemp phenology is its sexual dimorphism. Hemp is naturally dioecious, with the unisexual male and female flowers located on separate plants. Male and female plants do not flower and age simultaneously, with the male plants usually flower earlier (protandry) and age earlier. Since male flowers do not produce seed, a high frequency of male plants in the crop will reduce the seed yield. Male plants are also more susceptible to pests but have a finer fiber which is an advantage for application in textile manufacture. The development of stable monoecious cultivars (hermaphrodite plants, carrying male and female flowers on the same plant) is an important breeding goal. Compared to dioecious cultivars, the monoecious cultivars are more uniform in plant height, stem and seed production (Borthwick and Scully, 1954; Lisson et al., 2000a,b; Amaducci et al., 2008a,b, 2012; Salentijn et al., 2015; Small, 2015). However, while monoecious hemp is better for dual harvest of fiber and seed, it is considered that dioecious genotypes are superior for fiber production. There is evidence for genotypic variation in fiber quality, but due to the large variability in fiber characteristics created by environment and large influence of fiber extraction methods, the identification of varieties with specific fiber qualities is very difficult (Berenji et al., 2013; Amaducci et al., 2015).

Knowledge on the typical phenology of hemp is a prerequisite for successful hemp production and breeding for optimal combinations of flowering-time and fiber quality in a specific environment. Many aspects of hemp flowering are reviewed in detail by Hall et al. (2012). First of all, the development stage of hemp must be carefully monitored to determine the right moment for harvesting biomass for different products, and flowering-time is a main indicator for this. Around the onset of flowering, the flow of nutrients is shifting more to the development of flowers and seeds, and less to the development of stems, leaves, and roots, creating a change in carbon partitioning. Regarding fiber hemp, stem yield shows the highest increase before the onset of flowering and is positively correlated to the duration of the vegetative phase, and this is a reason why late-flowering cultivars with a prolonged vegetative phase produce the highest stem biomass (Höppner and Menge-Hartmann, 2007; Faux et al., 2013; Hall et al., 2014; Tang et al., 2016). The time of maximal stem, bark, and fiber yield (“technical maturity”) is reached at full (male) flowering (Mediavilla et al., 2001). Flowering time also marks the time-point of secondary fiber formation, from the bottom-upward in the stem. At the flowering stage, the lignification process continues and intensifies (Keller et al., 2001; Liu et al., 2015), accompanied by a decrease in cellulose and pectin deposition with plant maturity (Liu et al., 2015). This situation results in a proportional decrease in the primary bast fiber layer and increase in secondary bast fiber fraction along the stem. Thus, the quality of the fibers is influenced by the developmental stage at harvest and it differs between different sections of the stem. Based on fiber quality measurements, the best quality fibers are obtained from the middle part of stems, harvested around flowering (Keller et al., 2001; Mediavilla et al., 2001; Li et al., 2013; Liu et al., 2018). In addition to this, the after-harvest process “field retting” is important for a good separation of bast fibers from the woody core (shives). This process also influences the fiber properties such as the color, cellulose content, and crystallinity of the fibers (Mazian et al., 2018). The decision of harvest date should therefore also be determined on the basis of the optimal weather conditions for field retting (not extremely wet or dry). For the dual production of seed and fiber, harvest takes place at seed maturity, resulting in an increased proportion of more lignified and shorter secondary fibers (Amaducci et al., 2015).

A great wish of breeders is to gain more control over the phenology and fiber quality of hemp in order to breed for varieties with specific combinations of flowering-time and seed and fiber qualities. Current hemp cultivars still contain levels of genotypic and phenotypic heterogeneity in sensitivity to photoperiod and stability of monoecy, of which the outcome in different environments is hard to predict. In this respect, the complex phenological traits “flowering-time” and “sex determination” are very important traits to consider in fiber hemp breeding. However, currently there is only little molecular information available for hemp. Here we evaluate current knowledge on genetic components for fiber quality, flowering-time control, and sex determination in hemp and in other crops, relevant to hemp. Candidate genes for phenology and fiber quality in hemp are proposed and the prospects for using this knowledge for hemp breeding are discussed.

## Genetic Factors Involved in “Flowering-Time”: Learning From Other Crops

How are plants capable of sensing changes in their living environment, and how is the plant capable of responding to changing environments to ensure the most efficient timing of flowering?

Many studies, on multiple crops, have focused on the results of exhaustive analysis of the numerous genes involved in complex flowering-time gene networks in the model crops *Arabidopsis thaliana* (long-day plant) and rice (short-day plant), that are accessible *via* interactive databases (Bouché et al., 2016, http://www.flor-id.org, wikipathways: WP2312 (*Arabidopsis*) and WP2178 (rice)). From these studies, a complex picture emerged, whereby flowering is precisely controlled by cross-talk between multiple signaling pathways combining environmental and endogenous factors. This regulation network enables the plant to reproduce in changing environments.

The knowledge of flowering-time control is continuously expanding to other species, a broader range of flowering-related traits, such as yield and stress components, and specific allelic functionalization of flowering-time regulatory genes (Jung et al., 2017). The main pathways regulating flowering-time are: (1) the photoperiodic pathway, induced by variation in day length, (2) the gibberellic acid (GA)-dependent pathway, (3) the autonomous pathway, governed by the plant’s physiology status, independent of day length, (4) the vernalization and temperature pathway, induced by cold/ambient temperatures, (5) the aging pathway, induced by developmental factors that render the plant competent to flowering, and (6) the sugar pathway, in which the sugar status of the plant plays a role (e.g., Cho et al., 2018).

From studies in model crops, it is known that in the regulation of flowering-time, specific key genes are acting at distinct stages. The first stage is the perception and transduction of external signals; secondly, environmental and endogenous signals are transferred to special nodes in the signaling pathway; and thirdly, downstream “integrator genes” confer the capacity to flower to the meristems by the activation of floral meristem identity genes.

Hemp flowering is extremely sensitive to changes in photoperiod and temperature, and therefore the “photoperiodic pathway” and the “temperature pathway” seem to play prominent roles in regulating flowering-time in hemp.

The first stages in the “photoperiodic pathway” are the perception of light by photoreceptors (Smith, 2000; Jenkins, 2014; Christie et al., 2015; Galvão and Fankhauser, 2015; Xu et al., 2015; Kong and Okajima, 2016) and signal transduction to the central node of the “photoperiodic pathway” (the “GI-CO-FT” signaling cascade). The nuclear transcription factor CONSTANS (CO) acting in this node is essential for the induction of expression of *FLOWERING LOCUS T* (*FT*), coding for the mobile flower-promoting signal. *CO* expression is temporally regulated by the “circadian clock” gene *GIGANTEA* (*GI*), which has many other regulating functions in plant development, synchronizing genes in a 24-h daily rhythm (e.g., Valverde et al., 2004 (*CO*); Wenkel et al., 2006 (*CO*); Mishra and Panigrahi, 2015 (*GI*)). The next steps are performed by downstream acting “integrator genes” such as *FLOWERING LOCUS T* (*FT*), *FLOWERING LOCUS C* (*FLC*), and *SUPPRESSOR OF OVEREXPRESSION OF CONSTANS1* (*SOC1*) (for references, see Immink et al., 2012).

One of the most intriguing integrator genes involved in flowering is *FLOWERING LOCUS T* (*FT* or florigen; member of the *CETS* gene family). FT is the mobile flower-promoting signal that is transported over long distance from the leaf to meristems elsewhere in the plant to induce flowering (for references, see e.g., Notaguchi et al., 2008; Wigge, 2011). It is now clear that specific members of the *CETS* gene family have antagonistic functions in maintaining vegetative growth (indeterminate growth) or promote flowering (determinate growth), indicating that flowering is regulated by balancing between family members (McGarry and Ayre, 2012; Lifschitz et al., 2014). FT originates from specific phloem companion cells of leaf veins (Chen et al., 2018). After loading to the phloem, FT is transported to meristems where it promotes the transition from vegetative growth to flowering, together with other “integrator genes,” by regulating floral meristem identity genes which code for transcription factors such as LEAFY, APETALA1, AGAMOUS-LIKE 24, and FRUITFULL) (e.g., Lee and Lee, 2010).

An example of a successful approach to model flowering is the specific control of *FT* expression by transgenic ectopic overexpression or inactivation of *FT*. This approach was applied in angiosperm trees such as poplar, apple, citrus, and in a variety of woody and herbaceous species to induce precocious flowering (early flowering, shortening the juvenile stage), to facilitate an intermediate step that accelerates the breeding process (for ref, see Klocko et al., 2016). However, it has to be considered that in most crops, FT is encoded by small gene-families, with different functionalities and expression profiles among the members, within and among species. While some *FT* family members stimulate flowering, others may have the opposite function and inhibit flowering. Examples are reviewed by Wigge (2011), such as the case of sugar beet (*B. vulgaris*), where two *FT-like* genes are expressed, an activator and a repressor of flowering, and the case of tomato (*L. esculentum*) where one of the *FT* genes was not functioning on flowering but influenced leaf maturation, stem growth, and the formation of abscission zones. In such a situation, careful selection of specific candidate *FT*-loci is required. An example for the targeted inactivation of a specific *FT* gene, leading to delayed flowering, can be found in soybean. In soybean, ten *FT* homologs have been found and two of them are confirmed to control flowering (*GmFT2a* and *GmFT5a*). Cai et al. (2018) used the CRISPR/Cas 9 system to specifically knock out *GmFT2a* in soybean, resulting in truncated nonfunctional proteins. The mutations were stably inherited to the next generations and several homozygous, transgene clean lines without signs of off-target activity were selected. These induced mutants displayed, as expected, late flowering under both SD and LD conditions. However, besides *FT*, also other genes in the flowering-time pathway could affect growth as was observed in a late-flowering, double loss-of-function mutant (*soc1 fruitfull*) that displayed a woody phenotype (Melzer et al., 2008).

FLOWERING LOCUS C (FLC) mainly integrates signals from ambient temperatures, vernalization and autonomous signals. In many plant species, a period of cold temperature is required in order to promote flowering in the following spring (vernalization). FLC is a floral repressor and, in *Arabidopsis*, a period of low temperature is needed to release this inhibition (Michaels and Amasino, 1999; Bouché et al., 2015; Cheng et al., 2017). *FLC* genes appeared to have been lost in several lineages of flowering plants (Ruelens et al., 2013). In hemp, the influence of temperature is especially important during the juvenile stage (basic vegetative stage). Hypothetically, it is possible that *FLC-like* genes, if expressed in hemp, are involved in timing the completion of the basic vegetative phase that requires a certain temperature input, before entering the photoperiod-sensitive phase. Initiation of hemp growth requires a base air temperature of around 1°C, with an optimal temperature for growth of 29°C and a ceiling temperature 41°C. For completion of the basic vegetative stage and floral initiation, temperature degree days in the range 306–636°Cd are required (Amaducci et al., 2008b, 2012).

The third main integrator gene *SUPPRESSOR OF OVEREXPRESSION OF CONSTANS1* (*SOC1*) is a MADS-box transcription factor that, together with another transcription factor (AGL24), directly can activate the floral meristem identity gene *LEAFY* (*LFY*). *SOC1* acts in response to signals from multiple pathways; FLC (acting in the vernalization pathway) can bind to the promoters of *SOC1* and *FT* to repress their expression (Li et al., 2008), *SOC1* is indirectly regulated by *CONSTANS* (*CO*) *via* the levels of FT (photoperiod pathway), *SOC1* expression is regulated *via* the “aging pathway” *via* a squamosa promoter-binding-like transcription factor (SPL9) and microRNA156 (see below), and SOC1 integrates the “gibberellic acid-dependent pathway” *via* a yet unclarified mechanism (for more information Lee and Lee, 2010; Immink et al., 2012; Hyun et al., 2016).

## Natural Genetic Variation in Flowering-Time

Hemp adapts strongly to the growing season of a given region and therefore it is important to grow the right cultivar for the desired yield, fiber, seed, or both. Also, the maturity of the crop at harvest has a strong influence on the fiber quality. As a general rule, late dioecious cultivars are good for fiber production and early cultivars better for seed production. For dual harvest of fiber and seeds, monoecious early- or mid-early flowering cultivars are advised (e.g., Faux et al., 2013; Amaducci et al., 2015; Tang et al., 2016). A range of cultivars is already available but there is still need for improvement, for instance by breeding varieties with specific fiber qualities, lower lignin content, lower pectin content, altered architecture or higher seed yield and quality, in combination with specific flowering-time (early-, mid-, late-flowering for specific environments) and sex determination (monoecious or dioecious) (Hall et al., 2012; Salentijn et al., 2015).

Early flowering, under noninductive (LD) conditions and reduced sensitivity to photoperiod is interesting for latitudinal adaptation of hemp and pivotal for reproductive success and good fiber and seed yields in Northern latitudes. Late flowering, with a prolonged vegetative stage is used for adaptation to low latitudes to obtain more yield.

In many crops, genetic variation in specific genes has been employed for the development of selection markers to speed up breeding of cultivars adapted to more extreme environments. Because flowering-time is a complex trait, characterization of flowering-time pathways and the specific natural allelic variation underlying genetic loci correlated to quantitative traits (QTLs) for flowering-time, the development of molecular markers for flowering behavior in hemp is needed. If we consider short-day crops like hemp, examples for the molecular control of flowering-time can be found in the monocot rice (*Oryza sativa*) and the dicot legume soybean (*Glycine max* (L.) Merr.).

In the short-day plant rice, natural variation in *FT* homologs is a characteristic for more day-neutral flowering. The main *FT* homolog in rice, *Heading date 3a* (*Hd3A*)(Kojima et al., 2002), is upregulated in SD photoperiods and inhibited in LD, to allow flowering only in a SD regime and the central regulation node of flowering *Arabidopsis* (*GI-CO-FT*) is conserved in rice (*OsGI-Hd1-Hd3a*). *Hd3a* and its close homolog *RICE FLOWERING LOCUS T 1* (*RFT1*) are essential for flowering under SD conditions (Komiya et al., 2008). In specific rice cultivars that were selected for early flowering in LD conditions, *RFT1* is the major floral activator under LD conditions (Komiya et al., 2009). A rice ortholog of *AtSOC1*, *OsMADS50* (Ryu et al., 2009) and *Ehd1* (Doi et al., 2004) are positive regulators of *RFT1* while *Hd1* (Hayama et al., 2003), *phyB* (Dehesh et al., 1991), *Ghd7* (Xue et al., 2008), *Ghd8*, and *PRR37* (Yano et al., 2001) delay flowering under long-day conditions resulting in increased plant height, grain number per panicle, and grain yields (for references, see Komiya et al., 2009; Zhang et al., 2015; Hill and Li, 2016). Combinations of functional and nonfunctional alleles of these floral suppressors contribute to early flowering in LD and adaptation of rice to specific climates (Xue et al., 2008; Zhang et al., 2015).

In soybean, several natural variants controlling flowering and seed maturity time have been used in breeding for adaptation to the more Northern regions with longer periods of LD. These are mainly variants in the *E* genes (*E1* to *E10*), the *juvenile* (*J*) gene, and *FT* genes (*GmFT2a*, *GmFT5a* and *FT4*). The *E1* gene (which is a legume-specific transcription factor) (Xia et al., 2012) and the *FT4* gene (Samanfar et al., 2017) both act as floral repressors by downregulating *GmFT2a* and *GmFT5a* in LD conditions. *E2* is an ortholog of GIGANTEA (Watanabe et al., 2011), whereas *E3* and *E4* encode *phytochrome A* genes (respectively *GmPhya3* and *GmPhya2*)(Liu et al. (2008); Watanabe et al. (2009)) that integrate red to far-red ratios. The genes underlying loci *E5* to *E8* have not been identified yet, *E9* is *GmFT2a* (Zhao et al., 2016), and *FT4* is a candidate gene for the *E10* locus (Samanfar et al., 2017). Plants carrying loss-of-function alleles for *E1* to *E4* lead to photoperiod insensitivity by allowing higher expression levels of the *FT* genes and promoting flowering under long-day conditions (for more references, see Zhai et al., 2015; Zhao et al., 2016; Copley et al., 2018).

## Transition to the Reproductive Stage and Bast Fiber Quality of Hemp

Transition to the adult, reproductive phase is an important moment. Around the onset of flowering, nutrient flow and carbon partitioning is shifted to the development of flowers and seeds, and less to the development of stems, leaves, and roots. In hemp, the transition from the vegetative to the adult phase can be recognized by a change in the leaf arrangement from opposite to alternate, the formation of inflorescences (Hall et al., 2012), a reduction in stem growth (Faux et al., 2013; Tang et al., 2016), and formation of secondary more lignified bast fibers from the bottom-upward in the stem (Keller et al., 2001; Mediavilla et al., 2001; Li et al., 2013; Liu et al., 2015, 2018). Furthermore, Liu et al. (2015) observed a reduction in bast content and thickness of the primary bast fiber layer in stems with plant maturity, which was related to the development and ripening of the seeds. Bast fibers of hemp are bundles of phloem cells derived from the vascular bundles of stems, with primary bast fibers derived from the procambium and secondary bast fibers from the vascular cambium (Gorshkova et al., 2012). One way in which the quality of hemp fibers can be improved is to reduce the proportion of more lignified and shorter secondary bast fibers relative to primary bast fibers (Amaducci et al., 2015).

With the aim to identify key genes related to hemp fiber quality, Van den Broeck and co-workers (Van den Broeck et al., 2008) studied differential expression profiles of over 1,000 unique hemp genes in bast tissue versus the more lignified core tissue during development. They found that hemp genes acting in five interconnected metabolic pathways (pentose phosphate pathway, shikimate pathway, aromatic amino acid biosynthesis, lignin biosynthesis, and one-carbon metabolism) were upregulated in the lignified core tissue, suggesting a direct or indirect link with lignin. Most of these genes were also found to be expressed in the bast fiber tissue but at a much lower level. The relative expression levels of some lignin-related genes increased during development. For instance, a gene with homology to *caffeoyl-CoA O-methyltransferase* involved in lignin biosynthesis peaked in bast tissue at 91 days after sowing. More recently, Guerriero et al. (2017) performed annotation and transcriptional profiling (RNAseq) of over 3,000 transcript assemblies in bast fiber tissues derived from different hemp stem sections. It was observed that in comparison to other stem sections, the transcriptome of the older internodes showed enrichment for phytohormone-related genes (e.g., genes involved in auxin metabolism, and gibberellic acid, abscisic acid, and jasmonic acid biosynthesis), together with genes involved in noncellulosic polysaccharide deposition and lignification. This is in accordance with a high degree of lignification in more mature fiber tissues at the bottom of the stems and gives many leads to candidate genes for further functional analysis (Table 3). The important role of jasmonic acid in the stimulation of secondary growth was strengthened by the observations of Behr et al. (2018b) who showed that exogenous application of jasmonic acid on young hemp plantlets stimulated the formation of additional secondary phloem fibers and enhanced the lignin content. Putative candidate hemp genes for the biosynthesis of monolignols, their oxidative coupling (laccases and class III peroxidases), lignin deposition (dirigent-like proteins), and stereo-conformation of lignans (dirigent proteins) were studied in more detail (on the gene expression and protein level) underpinning their putative functional relation to lignification of hemp bast fibers (Behr et al., 2018a).

In *Arabidopsis,* specific miRNAs (small non-protein coding RNA molecules; microRNA; miRNAs) in the leaves function as a signaling system that allows the plant to monitor the progress in development to adulthood and adequately help to time and to induce the flowering stage. This signaling operates *via* a balance between the amounts of two miRNAs, miR156 and miR172. From the juvenile to adult stage of *Arabidopsis*, a decrease in miR156 and an increase in miR172 in the leaves are observed. Expression of *miR156* can repress adult leaf traits and flowering by binding and inhibiting the genes coding for squamosa promoter-binding-like (SPLs) transcription factors that allow floral transition by activating *miR172*. In contrast, miR172 promotes flowering and adult leaf traits (Wu et al., 2009; Matsoukas, 2014). In the short-day crop soybean, *miR156* and *miR172* were shown to be regulated by photoperiod. Indeed, in soybean, lower miR156 levels (repressor of flowering) and higher miR172 (inducer of flowering) levels were observed under SD than LD photoperiods (Li et al., 2015; Sánchez-Retuerta et al., 2018). Interestingly, *miR156* members also function in the phenylpropanoid biosynthesis pathway, where a *miR156*-targeted *SPL9* has been shown to regulate the metabolic flux during flavonoid biosynthesis (Gou et al., 2011; Gupta et al., 2017). The expression of miRNA families (*csa-miR156*, *csa-miR159a*, *csa-miR171b*, *csa-miR172a*, *csa-miR5021a*, *csa-miR6034*) is *in silico* predicted in hemp (Das et al., 2015; Hasan et al., 2016). However, to date no further information is available on the regulatory patterns of specific miRNAs in hemp. An interesting research question would be if lignin and secondary cell wall deposition that occurs around flowering can be attributed to specific miRNAs. Due to the presence of lignification and high variation in flower characters, hemp seems to be an ideal model crop to study interactions between signaling pathways which is expected to result in the identification of efficient molecular tools to improve the fiber quality in hemp.

## The Diverse Roles of Gibberellic Acid and Other Phytohormones

Another player in the regulation of flowering-time is the phytohormone gibberellic acid (GA). Gibberellins are known to be required for normal growth and development in several species. In *Arabidopsis*, gibberellins are known to be involved in the “gibberellic acid (GA)-dependent flowering pathway” by regulating *AtSOC1* and the floral meristem identity gene *LEAFY* (*AtLFY*) (Moon et al., 2003; Mutasa-Göttgens and Hedden, 2009). The GA-dependent growth-regulated pathway is connected with the light regulatory pathway and the circadian clock *via* PHYTOCHROME INTERACTING FACTORS (PIFs). *PIFs* are regulated by light through phytochrome, and the interaction with the GA-regulated DELLA proteins can block their activity, and thereby suppress the ability of PIFs to promote gene expression and growth (Mutasa-Göttgens and Hedden, 2009; Leivar and Monte, 2014). Also, the biosynthesis pathway of gibberellins is regulated by several endogenous and environmental factors including light, developmental stage, and hormone balance (Hedden and Phillips, 2000). Changes in active GA levels that occur for instance in response to altering light intensities influence plant cell development and the cell wall composition (e.g., Falcioni et al., 2018). The enzyme GIBBERELLIN 20 OXIDASE (GA20 OX) is a key enzyme in the formation of bioactive GAs, whereas GIBBERELLIN 2 OXIDASE (GA2 OX) acts in the opposite way by inactivating bioactive GAs. Both enzymes were shown to modulate plant growth when modified genetically. Overexpression of *GA20 OX* in various plant species resulted in e.g., increased seed yields, biomass increase, longer xylem fibers, longer and larger leaves whereas knockdown of *GA2 OX* resulted in increased (tobacco) growth and fiber production (reviewed in de Lima et al., 2017). Transgenic tobacco plants expressing the *Arabidopsis* genes *GA20 OX* or *GA2 OX* show high or low GA levels, respectively, resulting in elongated or stunted pants, respectively. The effects on dry matter accumulation that were found among these transgenic tobacco plants were most likely due to changes in lignin deposition due to upregulation of genes acting in lignin biosynthesis at increased GA levels (Biemelt et al., 2004). Overexpression of *GA2 OX* in *Jatropha* and *Arabidopsis* induced dwarfs with smaller leaves, flowers, and fruits, with a late flowering effect observed only in the latter (Hu et al., 2017). In hemp, exogenous application of GA is used to induce male flowers on female plants, but male plants showed no change in sex determination when treated with GAs (Mohan Ram and Jaiswal, 1972). Exogenous application of GA on leaves increased the growth of hemp and the treated plants showed a greater number of fibers compared to controls. The individual fibers were larger in diameter, more lignified, and up to 10 times as long as the fibers from the untreated plants (Atal, 1961). Application of the phytohormone jasmonic acid to young hemp plantlets resulted in an increased secondary growth as well as the formation of additional secondary phloem fibers, increase in lignin deposition and upregulation of lignin-related genes (Behr et al., 2018b). Also, fibers in the bottom parts of hemp stems were enriched for the expression of genes involved in GA biosynthesis and the biosynthesis of other phytohormones (Guerriero et al., 2017) pointing at the involvement of phytohormones in the regulation of secondary fiber growth.

## Genetic Components of Sex Determination

Hemp has a diploid genome (2*n* = 20) composed of nine pairs of autosomal chromosomes and one pair of sex chromosomes. Like in human, the gender of hemp is known to be influenced by a XY chromosome system. The hemp males are always XY while females carry the XX karyotype (Ainsworth, 2000; Moliterni et al., 2004; Ming et al., 2011; Divashuk et al., 2014; Faux et al., 2014; Razumova et al., 2016). Monoecious hemp, with the female and male flowers located on the same plant, has generally the female XX karyotype (Faux et al., 2014). The key factors that are driving this sexual dimorphism are still unknown (Westergaard, 1958; Ainsworth, 2000; Matsunaga and Kawano, 2001; Ming et al., 2011). The sex determination seems to be more stable and definite in the male XY karyotype, showing the typical male morphology. However, the ability to develop male flowers on monoecious XX karyotypes shows that the male-determining and/or female-suppressing factors are not necessarily located on the Y chromosome (Faux et al., 2016). To identify sex-linked genomic sequences in hemp, linkage mapping has been performed (Mandolino and Ranalli, 2002; Peil et al., 2003; Faux et al., 2016). Faux and co-workers used populations, segregating for male and female plants, to map several sex-linked QTL loci, putatively located on sex chromosomes. Furthermore, groups of markers co-segregating with sex and with stability of sex determination were found (Faux et al., 2016). Comparison of gene expression (cDNA-AFLP) in early male and female apices resulted in the identification of several differentially expressed fragments, with homology to genes coding for a permease, a ubiquitin (SMT3-like protein), heavy chain of a kinesin 9 protein, and a Rac-GTP binding protein, which may be involved in auxin-regulated gene expression (Moliterni et al., 2004).

Regarding sex determination, an obvious similarity is found between spinach (*Spinacia oleracea*) and hemp. Like hemp, spinach is dioecious with occasionally monoecious plants in specific lines and crosses, but, in contrast to hemp, no heteromorphic sex chromosomes are observed (Ramanna, 1976). Sex determination in spinach is determined by a locus for sex determination carrying the Y and X alleles, whereas monoecy is controlled by a single, incomplete dominant gene on the M locus that is closely linked to the X/Y locus, as was determined in a specific breeding line (Yamamoto et al., 2014). In the presence of the incomplete dominant M allele, female plants (XmXm and XXm) are monoecious whereby the homozygous XmXm plants show a higher degree of male flowers compared to the XXm plants (M masks X) and, because Y is dominant over X and M, YX and YXm plants are male. So, M is a male-promoting, female-suppressing factor but is less effective than the Y allele. In spinach, monecious lines are used for breeding because of the high degree of homozygosity and high-male monoecious lines are wanted therefore as male parents in breeding programs (Yamamoto et al., 2014). Also, in both spinach and hemp, gibberellins promote masculinization. Recently, West and Golenberg (2018) studied the role of gibberellic acid signaling (GA) in sex determination of spinach and came up with an interesting model for the action of genes underlying sex determination in spinach. They observed differential expression of the *GIBBERELLIC ACID INSENSITIVE* gene (*SpGAI*), which is a transcription factor of the DELLA family, among female and male inflorescences, with a high *spGAI* expression observed in female inflorescences. Based on gene function analysis studies, a signaling pathway toward sex determination was proposed in reaction to GA application. In short: high levels of GA inhibit *SpGAI*. GAI inhibits the expression of spinach B-class homeotic genes, which are masculinizing factors that stimulate male organ formation and at the same time suppress the development of female organs in flower primordia. So, in conditions of high GA levels (external GA application), the GAI content is reduced, resulting in the release of the inhibition on the B-class homeotic genes, formation of male organs, and inhibition of female organ development. Indeed, in female inflorescences, a two-fold higher expression of *SpGAI* was observed compared to male plants, which is in agreement with a higher GAI content leading to female organ development.

## Pleiotropic Effects and Carbon Partitioning

Variation in flowering-time is often linked to variation in developmental traits such as plant height, ear height (in maize), seed yield, seed quality traits, leaf number, cell wall composition, and secondary growth [Melzer et al., 2008 (*Arabidopsis*); Durand et al., 2012 (maize); Vanous et al., 2018 (maize); Cober and Morrison, 2010 (soybean); Shen et al., 2018 (*Brassica napus*); Copley et al., 2018 (soybean); Petit et al., in preparation (hemp)]. Members of the *FT* gene family may be involved in these pleiotropic effects (see above), but also other genes operating in signaling networks may connect flowering traits with development and growth. For instance, based on expression network profiling using a late-flowering, woody double mutant (*soc1ful*) of *Arabidopsis* (Melzer et al., 2008), three genes with dual function in growth and flowering were indicated as potential candidates for the link between the flowering pathway and growth (*XAL1*, *AN3*, and *REM1*) of which one, *AN3*, has FT-like properties (Davin et al., 2016).

This correlation of traits complicates selection procedures since negative co-effects on traits have to be considered. An example is soybean, where earliness is often accompanied by a loss in seed yield and quality. To examine these pleiotropic side effects of early flowering, a series of isogenic soybean lines carrying “photoperiod insensitive alleles” (at loci E1, E2, E3, E4, and E7 for early flowering, under LD, see above) was monitored for multiple agronomic traits. The whole series of isogenic lines, including lines with mutations in multiple loci, provided a range of flowering-times, maturities, and yields. For isogenic lines with a single mutant locus, early flowering was often associated with shorter plants, reduced lodging, and early maturity but unfortunately also with reduced seed yields. Among the lines with multiple mutations, some interesting lines with zero yield reduction were found, which might be due to additive or epistatic effects of combined alleles (Cober and Morrison, 2010). In a search for novel loci and genes for photoperiod insensitivity and maturity in soybean, Copley et al. (2018) performed genome-wide association studies (GWAS) and identified several novel loci for maturity traits. However, as most traits were correlated, also most QTLs were co-localized. This correlation of phenotypes can be explained by either a clustering of several genes in a locus or by a “pleiotropic effect” of a single gene on several traits.

Such correlated changes in phenotypic patterns may reflect the shifts in carbon partitioning that take place during development, which affect the overall plant morphology. In earlier varieties, less biomass is accumulated in stem and leaves and therefore less carbon is available for seed production. Interestingly, overexpression of an *AGAMOUS-like* MADS-box transcription factor, *GmAGL1*, induced early flowering in soybean, but without negative effects on seed production or on oil and protein content in seeds (Zeng et al., 2018). The only pleiotropic effect of earliness in these transgenic lines was that they had smaller petals and shortened inflorescences. Based on this, it was hypothesized that the transgenic plants may compensate for the energy required for developing fruiting organs by reducing a further allocation to vegetative organs (shortened inflorescences and slightly reduced growth of petals).

In *Arabidopsis*, it was shown that the shift in carbon partitioning during development is tightly controlled and involves the action of sucrose transporters (SUTs), hexose transporters (STPs) that function in uptake to a cell, and SWEET transporters for export out of the cell, as well as sucrose cleavage enzymes such as cell wall invertases (CINs), vacuolar invertases (VINs), and sucrose synthases (SUSs). In addition to their multiple functions, including acting as energy source (sugars), storage molecules (starches) and structural components (fibers), carbohydrates can also act as signaling molecules (Cho et al., 2018 and references therein). Trehalose-6-phosphate (T6P) and Hexokinase 1 (HXK1) are such important signaling metabolites, regulating carbon assimilation and sugar status in plants. At flower induction, sugar consumption for growth reduces and the remaining glucose that is accumulating in the phloem of leaves can eventually promote expression of florigens, while trehalose-6-phosphate functions in the shoot apical meristem to promote the flowering signal pathway downstream of those florigens (Ponnu et al., 2011; Matsoukas, 2014; Cho et al., 2018).

In hemp, the upregulation of genes acting in lignin biosynthesis in older bast fibers (Guerriero et al., 2017) may reflect carbon partitioning toward lignin biosynthesis in phloem tissues around flowering.

## A Candidate Gene Approach Toward Genetic Control of Hemp Phenology and Fiber Quality

Flowering-time, sex determination, and fiber quality of hemp are quantitative traits that are governed by many genetic loci, each with a certain effect on the phenotype in a specific environment or at a certain developmental stage, or in general. A “candidate gene approach” can contribute to the knowledge about traits. The identification of biosynthesis and signal routes that play a role in the traits enables the identification of candidate genes with the greatest effects on the downstream phenotype, and the prediction of pleiotropic effects on other traits. A selection of genes that are hypnotized to have profound effects on phenology and bast fiber quality in fiber hemp are shown in Tables 1–3.

**Table 1:** A selection of candidate genes for controlling flowering-time in the short-day crop hemp.

Candidate gene	Protein description/ortholog	Function (species)	Reference
*E2*	GIGANTEA	Photoperiod sensitivity (Soybean)	Watanabe et al., 2011
*GmPhyA2* *GmPhyA3*	Phytochrome A	Photoperiod sensitivity (Soybean)	Liu et al. (2008) Watanabe et al. (2009)
*GmFT2a, GmFT5a*	FLOWERING LOCUS T	Promoting flowering (Soybean)	Kong et al., 2010
*GmFT4* and *E1*	FLOWERING LOCUS T	Repressors of *GmFT2a* and *GmFT5a* in LD (Soybean)	Samanfar et al., 2017 Xia et al., 2012
*J*	EARLY FLOWERING 3	Relieving the suppression of FT expression by E1; loss of function alleles show delayed flowering (Soybean)	Lu et al., 2017
*HD3A*	HEADING DATE 3A FLOWERING LOCUS T	Promotes flowering in SD (Rice)	Monna et al., 2002 Kojima et al., 2002
*RFT1*	RICE FLOWERING LOCUS T 1	Promotes flowering in SD and LD (Rice)	Komiya et al., 2008, 2009
*OsMADS50*	MADS-box transcription factor 50/AtSOC1	Promotes flowering in LD (Rice)	Ryu et al., 2009; Komiya et al., 2009
*EHD1*	Two-component response regulator ORR30	Promotes flowering in SD (Rice)	Doi et al., 2004
*HD1* *OsPhyB* *GHD7* *GHD8*/HD5 *PRR37*	Zinc finger protein HD1/CONSTANS Phytochrome B Transcription factor GHD7 Nuclear transcription factor Y subunit B-11 Two-component response regulator-like PRR37	Inhibition of flowering in LD (Rice)	Hayama et al., 2003 Dehesh et al., 1991 Xue et al., 2008 Yano et al., 2001 Yano et al., 2001
*FLC*	MADS-box protein FLOWERING LOCUS C, AGAMOUS-LIKE 25	Temperature-dependent flowering. Repressor of flowering (*Arabidopsis*)	Michaels and Amasino, 1999

LD, long-day photoperiod; SD, short-day photoperiod.

**Table 2:** A selection of candidate genes for sex determination, growth, and development in hemp.

Candidate gene	Protein description/ortholog	Function (species)	Reference
*GAI-like*	DELLA protein GAI	May inhibit B-class homeobox genes that promote male organ development. Upregulated in female inflorescences of spinach (Spinach)	Peng et al., 1997 Dill et al., 2001 West and Golenberg, 2018
*GID1*	Gibberellin receptor GID1	Gibberellin (GA) receptor; interacts with DELLA proteins in the presence of GA4 (Rice, *Arabidopsis*)	Nakajima et al., 2006 Griffiths et al., 2006 Ueguchi-Tanaka et al., 2005
*GA20OX*	Gibberellin 20 oxidase	Key oxidase enzymes in the biosynthesis of gibberellin (Rice, *Arabidopsis*)	Phillips et al., 1995 Rieu et al., 2008a
*GA2OX*	Gibberellin 2-beta-dioxygenase	Catabolism of biologically active gibberellins; GA homeostasis (Rice, *Arabidopsis*)	Thomas et al., 1999 Rieu et al., 2008b
*SPL*	Squamosa promoter-binding-like transcription factors	A family of plant-specific transcript factors that play crucial roles in the regulation of plantgrowth and development	Klein et al., 1996 Preston and Hileman, 2013 Liu et al., 2016

Genes involved in gibberellic acid signaling (GA) and DELLA transcription factors are interesting candidate genes for all three hemp traits: flowering-time, sex determination, and fiber quality, depending on the specific developmental stage and/or tissue where they are expressed.

**Table 3:** A selection of candidate genes for bast fiber quality in hemp.

Candidate gene	Protein description/ortholog	Function (species)	Reference
*WAT1*	WALLS ARE THIN	Auxin efflux transporter required for secondary wall formation in fibers (*Arabidopsis*); upregulated in bast fibers of older, thicker, and more lignified stem sections (Hemp)	Ranocha et al., 2010; Guerriero et al., 2017
*OMT1*	Flavone 3′-O-methyltransferase 1	Catalyzes the methylation of monolignols, the lignin precursors; upregulated in bast fibres of older, thicker, and more lignified hemp stem sections (*Arabidopsis*; Hemp)	Moinuddin et al., 2010; Van den Broeck et al., 2008; Guerriero et al., 2017
*CCoAOMT*	Caffeoyl-CoA O-methyltransferase 1	Synthesis of feruloylated polysaccharides; upregulated in bast fibres of older, thicker, and more lignified hemp stem sections (*Arabidopsis*; Hemp)	Do et al., 2007; Guerriero et al., 2017
*NAC* *MYB4*	MYB and NAC domain containing protein	Involved in lignin biosynthesis; several are upregulated in bast fibres of older, thicker, and more lignified hemp stem sections (Hemp)	Zhao and Dixon, 2011; Guerriero et al., 2017; Behr et al., 2018a
*DLP4* *DLP5*	Dirigent-like proteins	Putatively involved in lignin deposition (Hemp)	Behr et al., 2018a
*IRX12*	Laccase4	Oxidative coupling of monolignols (H, G, S-units) (*Arabidopsis*); upregulated in bast fibres of older, thicker, and more lignified hemp stem sections (Hemp)	Brown et al., 2005; Zhao et al., 2013; Guerriero et al., 2017; Behr et al., 2018a
*LOX2* *4CLL7*	Lipoxygenase 24 Coumarate CoA ligase-like 7	Jasmonic acid biosynthesis (*Arabidopsis*; Hemp)	Bell and Mullet, 1993; Schneider et al., 2005; Guerriero et al., 2017; Behr et al., 2018b

Genes involved in gibberellic acid signaling (GA) and DELLA transcription factors (see Table 2) are interesting candidate genes for all three hemp traits: flowering-time, sex determination, and fibre quality, depending on the specific developmental stage and/or tissues where they are expressed.

Hemp orthologs for genes acting in flowering-time signaling pathways are putative candidate genes for the regulation of flowering-time in the SD plant hemp. A selection of promising candidates is shown in Table 1.

The most obvious candidates are orthologs of the “*GI*-*CO*-*FT*” core genes of the “photoperiodic pathway” and genes coding for the phytochrome receptors. In other SD crops, early flowering was often observed in plants carrying nonsense mutations in genes that are repressors of flowering in LD conditions (e.g., orthologs to soybean *E1* to *E4* genes, (Langewisch et al., 2017) and orthologs of rice *Ghd7*, *Ghd8*, *PRR37,* and *phyB* (e.g., Xue et al., 2008). Later flowering can for instance be found in plants with nonsense mutations in the florigens or other genes that stimulate flowering (Cai et al., 2018).

When considering a candidate gene, one has to take into account that many genes acting in the flowering-time signaling networks have pleiotropic effects on other traits or may belong to a gene family of which the individual family members have different functions. For instance, the “circadian clock” genes such as *GI*, and other “circadian” genes involved in regulation of flowering-time are involved in many biological processes, and can result in pleiotropic effects on for instance floral transition, leaf movement, stomata opening, seed germination, and hypocotyl elongation (e.g., Ding et al., 2007; Kolmos et al., 2009; Wenden et al., 2011; Mishra and Panigrahi, 2015; Shim and Imaizumi, 2015). Also, squamosa promoter-binding-like proteins (SPLs) belong to a family of functionally specialized transcription factors with multiple roles in plant phase transition, flower and fruit development, plant architecture, gibberellins signaling, sporogenesis, and response to copper and fungal toxins (Preston and Hileman, 2013).

Regarding sex determination, genes involved in gibberellic acid signaling (GA) and *DELLA* transcription factors are interesting candidate genes that may also have a side effect on fiber quality (Tables 2, 3).

In a situation where different quantitative characteristics have to be combined, the ability to select in an early stage for plants with specific flowering characteristics would already be an important step for breeding. Genetic variation at candidate gene loci can be utilized to select specific haplotypes *via* “haplotype tagging SNPs” (htSNPs). These htSNPs improve the efficiency of association studies performed for the selection of alleles in the population that are associated with phenotypic variation in the trait (Ehrenreich et al., 2009). In short-day crops such as soybean and rice, molecular markers for maturity and flowering-time based on genetic variation in candidate genes for flowering-time are already used (e.g., Langewisch et al., 2017 (soybean); Shabir et al., 2017 (rice)). In hemp, molecular markers have mostly been developed for forensic studies to differentiate drug-type cannabis from hemp or for the early detection of male plants (Mandolino et al., 2002; reviewed in Onofri and Mandolino, 2017).

It should also be stressed that finding “candidate genes” across species has limitations because the function of candidate genes across species may be similar but often not identical (e.g., Salentijn et al., 2007; Wong et al., 2014). So, a prerequisite for a successful application of the “candidate gene approach” is functional knowledge of candidate genes in hemp. At present, the knowledge of gene function and gene expression in *Cannabis sativa* is still limited, and mainly focused on genes acting in cannabinoid biosynthesis. Several molecular technologies such as “genome editing” and “targeted mutagenesis” contribute to gene functional analysis and to the generation of plants with specific mutations. Genome editing by the CRISPR/Cas9 system appears to be a very precise and efficient tool for functional analysis of specific genes and the development of useful mutants in several crops (e.g., Hille et al., 2018; Schindele et al., 2018). The system yet requires genetic transformation and regeneration of transgenic CRISPR/Cas9 plants from undifferentiated cells (callus tissue) or protoplasts. Regarding hemp, protocols for shoot regeneration from callus are known, but these work efficiently only for specific hemp accessions (Andre et al., 2016; Chaohua et al., 2016) and, to our knowledge, cases of efficient production of transgenic hemp plants produced *via Agrobacterium*-mediated genetic transformation have not been published yet. As such, hemp is still considered a recalcitrant plant for genetic modification and thus for CRISPR/Cas9.

If mutants can be obtained in a less recalcitrant hemp cultivar, the specific mutations in such transgenic lines can be delivered to breeding lines *via* cross breeding. However, this will introduce also unwanted traits and the Cas9-gRNA cassette in the receiving parent, and many subsequent breeding steps are required to restore original traits. Recently, Kelliher et al. (2019) and Wang et al. (2019) published a new approach (Haploid-Inducer Mediated Genome Editing) to overcome such problems in maize cultivars. This approach combines the technology of haploid induction with CRISPR/Cas9 genome editing and requires specific haploid inducer lines (e.g., carrying homozygous mutations in *CENH3* for dicots) that are stably transformed with constructs expressing the CRISPR Cas9-gRNA editing tools. The gametes of such lines can transfer the editing tools to recalcitrant cultivars (*via* cross breeding instead of genetic transformation). Due to the haploid inducer, the genome carrying both, the CRISPR sequences and the haploid inducer trait is eliminated short after fertilization and haploid embryos of are formed that, upon chromosome doubling, can grow into plants that yield 100% inbred seed. It appeared that the short time of interaction of the two genomes after fertilization was enough to induce specific mutations in the recipient genome. Above all, the CRISPR genes are not present in genome of the resulting crop which can be advantageous in connection with GMO regulations. Application of this system in hemp is not to be expected in the short term since transformable hemp accessions, together with a haploid induction system, are not immediately available.

Targeted mutagenesis or TILLING (McCallum et al., 2000) is another way to select for plants with mutations in specific genes. For this strategy, seeds or pollen are treated with specific chemicals that make point-mutations at random throughout the genome. Large populations of mutated plants are then screened for the presence of mutations in specific genes using high throughput sequencing, or other screening technologies. This strategy was used in hemp to find plants with specific induced knock-out and missense mutations in *CsFAD2* and *CsFAD3* genes leading to altered seed-oil composition in the seed hemp variety Finola (Bielecka et al., 2014). Such a strategy requires facilities to grow large mutant populations and for seed storage and breeding steps to obtain homozygous mutations or combine different mutations.

A very useful tool for hemp genomics is the draft genome sequence of hemp (covering 534 Mb of the haploid hemp genome that is 818–843 Mb in size) published by Van Bakel et al. (2011), including more than 30,000 transcript assemblies (NCBI TSA: JP449145.1 to JP482359.1; PK00001.1 to PK29878.1), and the “*in silico*” gene expression profiles of these genes (Massimino, 2017). Two initiatives to improve the hemp genome were undertaken that independently resulted in the assembly of the hemp genome in 10 pseudomolecules (scaffolds, separated by gaps) representing the 10 different chromosomes of hemp (2*n* = 2*x* = 20) (Grassa et al., 2018; Laverty et al., 2018). It was experienced that the assembly of the hemp genome was complicated by the presence of large quantities of repetitive DNA (~73% of the hemp genome), the heterozygous character of hemp (Van Bakel et al., 2011; Sawler et al., 2015), and an expected high degree of karyotype polymorphisms among hemp varieties (Razumova et al., 2016). This situation was approached by using long-read sequencing technologies (PacBio SMRT, Nanopore sequencing) next to the standard Illumina sequencing technology to span large stretches of repetitive DNA. For the assembly of the genome, a combination of physical and genetic mapping was applied (Laverty et al., 2018). It was found that most recombination events occurred in the gene-rich regions near the chromosome ends. Furthermore, three pseudomolecules appeared to have recombination only on a single arm of the chromosome (telocentric) and one of these may represent the sex chromosome whereas the other two may represent the chromosomes that harbor 5SrDNA and 45SrDNA (Laverty et al., 2018). The map is still not completed (see NCBI assembly no. GCA_003417725.2 & GCA_000230575.4; GCA_900626175.1) and not all known transcripts and male-specific markers could be mapped. Dedicated genetic mapping and sequencing strategies may further unravel the complex genetic structure of the hemp genome, and may detect hemp lines that accommodate specific genetic variation.

## Concluding Remarks

Here we review aspects of the traits “flowering-time,” “sex determination,” and “fiber quality” that are relevant to hemp. This information can be utilized to predict putative candidate genes, which can serve as targets for the development of molecular markers for these traits. For the development of such breeding tools, it is important to know the allelic variation underlying candidate genes that is responsible for the phenotypic variation. A big advantage for hemp is the presence of a high level of natural genotypic and phenotypic variation which makes it possible to perform efficient GWAS studies to validate the putative biological function of candidate genes, and to discover novel genomic regions involved. Furthermore, we like to point to the importance of high throughput phenotyping protocols which are needed to map QTL loci, including small effect loci.

## Author Contributions

ES and LT were involved in the conceptualization of the review. ES wrote the manuscript. LT and JP revised the manuscript. All authors approved the manuscript.

### Conflict of Interest Statement

The authors declare that the research was conducted in the absence of any commercial or financial relationships that could be construed as a potential conflict of interest.
